# The “Campaign of Education”

**Published:** 1900-09

**Authors:** 


The "Campaign of Education."—The committee appointed at the last annual meeting of the Texas State Medical Association (Waco, April, 1900) to "compile, edit and publish, and distribute in pamphlet form the several papers and addresses on State medicine," etc., and the discussion of same, have been much hindered in their work, partly in consequence of defective and erroneous stenographic report made of same. The report was published in the two medical journals at Austin, and there were numerous complaints from those who participated in the discussion that they were incorrectly reported; hence, revised proof sheets were submitted to each one, and there was more or less delay in getting them back. Dr. Harrison, chairman of the committee, has been absent from home a good deal meantime, attending democratic convention at Waco and medical meetings in various sections of the State, and thus, while the matter has been in type since immediately after the meeting at Waco, the pamphlets are not yet out. It is hoped to have them ready for distribution during the present month. The Transactions, meantime, is under way, and the volume will be ready, we are informed, before October 1st. The Association's stenographer disappointed the Secretary, Dr. West, and all hands, by the very dilatory and negligent course he pursued with reference to forwarding the report of the meeting.

